# Homocysteine Serum Levels in Diabetic Patients with Non Proliferative, Proliferative and without Retinopathy

**DOI:** 10.1155/2014/191497

**Published:** 2014-04-28

**Authors:** Giulia Malaguarnera, Caterina Gagliano, Maria Giordano, Salvatore Salomone, Marco Vacante, Claudio Bucolo, Filippo Caraci, Michele Reibaldi, Filippo Drago, Teresio Avitabile, Massimo Motta

**Affiliations:** ^1^International Ph.D. programme in Neuropharmacology, University of Catania, 95123 Catania, Italy; ^2^Department of Ophthalmology, University of Catania, 95123 Catania, Italy; ^3^Research Center “The Great Senescence”, University of Catania, 95125 Catania, Italy; ^4^Section of Pharmacology and Biochemistry, Department of Clinical and Molecular Biomedicine, University of Catania, Viale Andrea Doria 6, 95125 Catania, Italy; ^5^IRCCS, Oasi Maria S.S.-Institute for Research on Mental Retardation and Brain Aging, 94018 Troina, Italy; ^6^Department of Educational Sciences, University of Catania, Via Teatro Greco 84, 95124 Catania, Italy

## Abstract

Homocysteine has been associated with extracellular matrix changes. The diabetic retinopathy is a neurovascular complication of diabetes mellitus and it is the leading cause of vision loss among working adults worldwide. In this study, we evaluate the role of homocysteine in diabetic retinopathy analyzing the plasma levels of homocysteine in 63 diabetic type 2 patients with nonproliferative retinopathy (NPDR), 62 patients with proliferative diabetic retinopathy (PDR), 50 healthy subjects used as control group, and 75 randomly selected patients.

## 1. Introduction

Type 2 diabetes is increasing in modern societies [1]. TDM2 is a metabolic disease characterized by elevation of blood glucose concentrations, lipid abnormalities, and vascular complications. Diabetes is a major cause of both microvascular (retinopathy, nephropathy, and neuropathy) and macrovascular diseases (cardiovascular diseases and nontraumatic lower extremity amputations), affecting, therefore, nearly every organ in the body.

Chronic exposure to elevate glucose and fatty acid concentrations can cause damage in different types of cells by a variety of mechanisms (glucolipotoxicity) [2, 3]. The diabetic retinopathy (DR) is a neurovascular complication of diabetes mellitus and it is the leading cause of vision loss among working adults worldwide. DR is a multifactorial progressive disease of the retina where the pathogenesis of the disease is extremely complex involving many different cells, molecules, and factors [4]. Hyperglycemia, hyperlipidemia, dysregulated hormones levels, and growth factors induce a cascade of biochemical and physiological changes leading to the neurovascular damage in the retina through oxidative stress, inflammation, and apoptosis [5].

Homocysteine is a sulfur containing amino acid derived from the methionine metabolism. Elevation in plasma homocysteine is common in the general population, particularly in the elderly [6, 7].

Several studies indicate that mild elevations of homocysteine in plasma are associated with an increased risk for occlusive vascular disease, thrombosis, and stroke [8–11].

There are few reports on the relationship between plasma homocysteine and diabetic retinopathy. The aim of our study was to evaluate plasma tHcy levels in diabetic patients with and without retinopathy in order to investigate the role of tHcy in the progression of the diabetic retinopathy.

## 2. Methods and Materials

### 2.1. Patients

This study was carried out on patients with type 2 DM regularly attending the outpatient clinic at Cannizzaro Hospital in Catania. We enrolled 175 diabetic consecutive patients (91 females and 84 males; mean age 65.2 ± 11.8 years; mean duration of diabetes 7.6 ± 5.4 years). The exclusion criteria included (1) patients who were already on lipid lowering drugs or glitazones; (2) females taking oral contraceptive pills or hormone replacement therapy; (3) familial hypercholesterolemia; (4) hypothyroidism; (5) patients with chronic liver disease; (6) patients with kidney disease. Assessment of DR was performed by ophthalmoscopy and or biomicroscopy through dilated pupils by a retinal specialist, and fluorescein angiography was obtained when indicated. Examination of the retina was done through dilated pupils to determine the level of nonproliferative DR or proliferative DR or diabetes without retinopathy. The DR is characterized by retinal microvascular signs that indicate the progression of the disease, from nonproliferative diabetic retinopathy (NPDR) to proliferative diabetic retinopathy (PDR), leading to macular oedema (DMO) and the commonest cause of blindness in diabetic patients. Group 1: the control groups were 80 randomly selected healthy subjects under 65 years of age (42 males and 38 females), aged 24–64 years (mean 44.6 ± 10.5 years) composed of blood donors and randomly selected volunteers working at the University of Catania; the group 2 is constituted by 75 randomly selected patients (35 males and 40 females), aged 30–85 years (mean age 60 ± 9.2 years), 50% of whom were institutionalized in Catania. According to the random selection criteria, no biochemical or hematological analysis were performed in groups 1 and 2.

### 2.2. Methods

Venous blood samples were drawn from patients and all examinations were performed at 8.00 h after an overnight fast. The samples were allowed to clot and serum was separated from the erythrocytes by centrifugation at 4°C and at 1500 × g for 15 min. Total cholesterol, triglycerides, and fasting plasma glucose were enzymatically measured (Roche/Hitachi 912 analyzer; Roche Diagnostics, Switzerland). Serum creatinine levels (upper reference limit 120 *μ*mol/l) were assayed with routine laboratory method. The intra- and interassay CVs were 0.9 and 2.7%, respectively. The fasting plasma glucose concentrations were assayed using the glucose-oxidase method with intra- and interassay CVs of 0.8% and 2.1%, respectively. Clinical chemistry tests were performed in the medical center laboratory using standard methods. Fasting blood samples were taken at enrolment from participants. The blood withdrawals were centrifuged at 2500 g for 15 min and plasma was separated and stored at −80°C (until analysis). A part of each sample was used in order to measure tHcy concentration, according to the method of Asaki and Sako. Fasting plasma levels of homocysteine were considered normal between 5 and 15 *μ*mol/l (Graeme and Eikelbaom). The intra- and interassay CVs were 1.4 and 3.2%, respectively. High density lipoprotein cholesterol (HDL-c) was measured enzymatically in the supernatant after precipitation of apolipoprotein B-containing lipoproteins by phosphotungstate/MgCl_2_. Low-density lipoprotein (LDL) level was calculated by using the Friedwald formula. Measurement of HbA1c was made by high-performance liquid chromatography (Menarini Diagnostics, Italy). Anthropometric measurements including weight, height, and waist and hip measurements were obtained using standardized techniques. Height was measured with a tape to the nearest centimeter. Subjects were requested to stand upright without shoes with their back against the wall, heels together, and eyes directed forward. Weight was measured with a traditional spring balance that was kept on a firm horizontal surface. Subjects were asked to wear light clothing and weight was recorded to the nearest 0.5 Kg. Body mass index (BMI) was calculated by using the following formula: weight (Kg/height (m^2^)). Waist circumference was measured by using a nonstretchable measuring tape. The subjects were asked to stand erect in a relaxed position with both feet together on a flat surface; one layer of clothing was accepted. Waist girth was measured as the smallest horizontal girth between the costal margins and the iliac crest at minimal respiration. Hip was taken as the greatest circumference (the widest protrusion of the hip) on both sides; measurements were made to the nearest centimeter. Waist-to-hip ratio was calculated by dividing the waist circumference (cm) by the hip circumference (cm). Examination of the retina was done through dilated pupils to determine the level of nonproliferative DR and proliferative DR, by qualified ophthalmologists (T.A; C.G.).

## 3. Statistical Analysis

The results are presented as mean ± standard deviation. The following two-tailed tests at the *P* ≤ 0.05 level of significance were used to evaluate the study; the Mann-Whitney *U* test was used in the case of two independent samples and Spearman's rank correlation coefficient test was used to test for univariate relationships between variables. In order to evaluate the independent effects of covariates on tHcy concentration, a stepwise multiple linear regression analysis was performed.

## 4. Results

In our study 62 out of 175 enrolled patients had DR (Table 1). Regarding diabetic management, 38 were on dietary treatment, 88 were on metformin treatment, and 49 were on insulin treatment.

### 4.1. Laboratory Parameters

Subjects with DR had higher glycated hemoglobin levels (*P* < 0.001) and fasting plasma glucose (*P* < 0.001) compared to both subjects with NPDR and without DR.

Significant differences were observed when comparing subjects with PDR versus subjects with NPDR in total cholesterol (*P* < 0.05), LDL (*P* < 0.001), and triglycerides (*P* < 0.05). The comparison between PDR and subjects without DR showed differences in LDL (*P* < 0.05) and triglycerides (*P* < 0.05) (Table 2(a)). Regarding creatinine levels, randomly selected patients showed significant differences compared to PDR and controls (*P* < 0.001); PDR showed differences compared to randomly selected patients (*P* < 0.001), healthy controls (*P* < 0.001), and diabetics without retinopathy (*P* < 0.05); NPDR showed differences compared to randomly selected patients (*P* < 0.05) and healthy controls (*P* < 0.001); controls showed differences compared to all other groups (*P* < 0.001); diabetics without retinopathy showed differences compared to PDR (*P* < 0.05) and controls (*P* < 0.001). For homocysteine levels, randomly selected patients showed significant differences compared to PDR (*P* < 0.001), NPDR (*P* < 0.001), and controls (*P* < 0.05); PDR showed differences compared to randomly selected patients (*P* < 0.001), NPDR (*P* < 0.001), healthy controls (*P* < 0.001), and diabetics without retinopathy (*P* < 0.001); NPDR showed differences compared to randomly selected patients (*P* < 0.001), PDR (*P* < 0.001), healthy controls (*P* < 0.001), and diabetics without retinopathy (*P* < 0.001); controls showed differences compared to randomly selected patients (*P* < 0.05), PDR (*P* < 0.001), NPDR (*P* < 0.001), and diabetics without retinopathy (*P* < 0.001); diabetics without retinopathy showed differences compared to PDR (*P* < 0.001) and controls (*P* < 0.001).

### 4.2. Sensitivity, Specificity, Predictive Value, and Odds Ratio

The values of sensitivity for both PDR and NPDR groups were 64% versus 63%; specificity was 70% versus 70%; predictive value of positive test was 69% versus 54%; predictive value of negative test was 65% versus 50%; efficiency of the test was 67% versus 51%; prevalence was 52% versus 51%. The odds ratio values were, respectively, 4.24 versus 1.16.

## 5. Discussion

Numerous factors may have an effect on progression of diabetic retinopathy. In our study higher plasma levels of homocysteine have been found in diabetic with proliferative diabetic retinopathy compared to both nonproliferative DR and diabetics without retinopathy. A previous study found moderate hyperhomocysteinemia to be a stronger cardiovascular risk factor in patients with type 2 diabetes than in nondiabetic subjects, suggesting that synergistic effects of diabetes and excessive circulating homocysteine accelerate the development of atherosclerosis [12]. Homocysteine is toxic to the vascular endothelium and therefore induces thrombosis and thus may play a role in aggravating the hypoxic state such as that seen in diabetic retinopathy by further closure of the capillary bed. An increase in plasma and in vitreous concentration of tHcy in proliferative diabetic retinopathy has been described [13–15]. Recent study indicates that DNA methylation is an important player in both DNA repair and gene stability. There is growing evidence that histone modification and DNA methylation play an important role in the development of DR. It has been suggested that the inactivation of DNA repair pathways, which leads to an increased mutation rate and chromosomal instability, can initiate and accelerate the proliferative process [16]. In patients with diabetes mellitus the odds ratio for hyperhomocysteinemia was 4.24 and 1.16 in PDR and NPDR, respectively. Increasing evidence suggested that the proliferation rate of cells would cause an elevation of circulating tHcy or an increase in the concentration of cells would deplete folate and inactivate the methionine synthase catalyzed remethylation reaction. This potential link between the microvascular changes that occur in diabetic retinopathy and hyperhomocysteinemia may be useful as a predictor for retinopathy. Diabetic retinopathy is one of the microvascular complications of diabetes which may not have symptoms in the early stages. Control of these complications depends on proper management and monitoring of retinal status and blood glucose levels after the early detection of retinopathy but may progress to a sight-threatening stage if left untreated. Homocysteine and diabetes increase oxidative stress and reduce nitric oxide formation and may cause endothelial dysfunction [17, 18]. Homocysteine enhances smooth muscle proliferation and affects the extracellular matrix. Thus elevated homocysteine level may act as a pathogenetic link or an instrument through which various risk factors may exert their deleterious effect on the promotion of diabetic retinopathy.

Thus, understanding and characterizing the tHcy role in the pathogenesis of diabetic retinopathy could help in identifying novel target to combat this blinding disease which is the major cause of blindness in adults.

## Figures and Tables

**Table 1:** Demographic characteristics of the study population.

Patients with type II diabetes mellitus (*n* = 175)	
Female/male	91/84
Age (years)	65.2 ± 11.8
Smokers/no smokers	88/87
BMI (Kg/m^2^)	26.1 ± 3.2
Waist circumference (cm)	94.1 ± 8.71
Hip circumference (cm)	96.7 ± 8.25
Waist-to-hip ratio	0.97 ± 0.08
Systolic blood pressure (mmHg)	138.6 ± 13.4
Diastolic blood pressure (mmHg)	81.8 ± 8.7

**Table:** (a)

Parameter	PDR 62	NPDR 63	Without DR 50
Cholesterol total (mmol/L)	6.11 ± 1.02	5.71 ± 1.04**	5.88 ± 1.07*
HDL (mmol/L)	1.39 ± 0.34	1.31 ± 0.33*	1.38 ± 0.37*
LDL (mmol/L)	4.35 ± 0.37	4.09 ± 0.31***	4.19 ± 0.30**
Triglycerides (mmol/L)	1.86 ± 0.62	1.56 ± 0.64**	1.58 ± 0.51**
Fasting plasma glucose (mg/dL)	172 ± 26	135 ± 28***	147 ± 24***
HbA1c (%)	8.8 ± 0.6	7.2 ± 0.7***	6.4 ± 0.8***

Comparison between PDR and other groups: *P* = NS*; *P* < 0.05**; *P* < 0.001***.

**Table:** (b)

	Randomly selected patients *n* = 75	PDR (*n* = 62)	NPDR (*n* = 63)	Controls (healthy subjects) (*n* = 80)	Diabetic without retinopathy (*n* = 50)
Creatinine (mg/dL)	84 ± 18.4^CE§§§†^	96 ± 16.2^∗∗∗D§§§††^	91.8 ± 15.7^∗∗A§§§†^	64 ± 12.4^∗∗∗CF†††^	87 ± 13.7^∗BD§§§^
Hcy (µmol/L)	10.2 ± 4.7^CF§§†^	18.2 ± 5.6^∗∗∗F§§§†††^	14.4 ± 6.7^∗∗∗C§§§†^	7.8 ± 6.4^∗∗CF†††^	12.1 ± 6.8^∗CD§§§^

Comparison between randomly selected patients and other groups: *P* = NS*; *P* < 0.05**; *P* < 0.001***.

Comparison between PDR and other groups: *P* = NS^A^; *P* < 0.05^B^; *P* < 0.001^c^.

Comparison between NPDR and other groups: *P* = NS^D^; *P* < 0.05^E^; *P* < 0.001^F^.

Comparison between controls and other groups: *P* = *NS* ^§^; *P* < 0.05^§§^; *P* < 0.001^§§§^.

Comparison between diabetic without retinopathy and other groups: *P* = NS^†^; *P* < 0.05^††^; *P* < 0.001^†††^.
